# Supplementary material for: Temporal single-cell analysis reveals age-associated delay in immune resolution after respiratory viral infection
Source: bioRxiv. 2025 Dec 17:2025.12.15.694321. Preprint. [Version 1] doi: 10.64898/2025.12.15.694321 (PMC12724688; doi:10.64898/2025.12.15.694321)

bioRxiv preprint doi: https://doi.org/10.64898/2025.12.15.694321; this version posted December 17, 2025. The copyright holder for this preprint (which was not certified by peer review) is the author/funder, who has granted bioRxiv a license to display the preprint in perpetuity. It is made available under aCC-BY 4.0 International license.

## Supplementary Figures

**Supplementary Fig. 1 Characterization and immune profiling of the lung in young and aged hosts following influenza virus infection.**

Young (2-3 months old) and aged (~24 months old) mice were infected with the same dose of mouse-adapted influenza virus A/PR/8/34 (PR8). Samples were collected at 0, 2, 9, 14, 30, and ~60 d.p.i.

A. Body weight loss curve.
B. Features used to define the general immune populations shown in Fig. 1C. Dot size and color intensity represents the percentage of cells and average expression level, respectively, in given genes (rows) and cell types (columns).
C. Module scores for selected pathways, displayed as a dot plot for the cell types defined in Fig. 1C. Dot size and color represents the percentage of cells and average expression level, respectively, in given cell types (rows) and pathways (columns).
D-F. Gating strategies for myeloid cells (D), αβ T cells (E), and invariant T cells (F) used in spectral flow cytometry.
G. Kinetics of plasmacytoid dendritic cells (pDCs) in the lung, quantified by spectral flow cytometry.

Data in (G) are pooled from at least three animals per data point. Each dot represents the mean value for that sample, with error bars filled in color. Statistical analysis was performed using two-way ANOVA. Results are shown to indicate whether the age of the mice served as a source of variation: ns, $p \geq 0.05$; *, $p < 0.05$; **, $p < 0.01$; ***, $p < 0.001$; ****, $p < 0.0001$.

**Supplementary Fig. 2 Quantification of αβ T cells using scRNAseq and high-dimensional flow cytometry.**

A. Kinetics of proliferating αβ T cells quantified from scRNAseq data (Fig. 2A), expressed as a proportion of all events passing quality control in Fig. 1C.
B. Kinetics of CD8+ αβ T cells quantified from scRNAseq (Fig. 2A), expressed as a proportion of all quality-controlled events in Fig. 1C.
C. Gating strategy for identifying $CD69^+PD\text{-}1^{hi}$ $CD8^+$ T cells (representing age-associated T cells) and $CD69^+CD103^+CD8^+$ T cells by spectral flow cytometry.
D. Bar graph showing normalized enrichment score of selected GSEA pathways generated from ranked differential expressed genes comparing two $T_{RM}$ clusters at 61 d.p.i.

bioRxiv preprint doi: https://doi.org/10.64898/2025.12.15.694321; this version posted December 17, 2025. The copyright holder for this preprint (which was not certified by peer review) is the author/funder, who has granted bioRxiv a license to display the preprint in perpetuity. It is made available under aCC-BY 4.0 International license.

E. Kinetics of $CD4^+$ αβ T cells quantified from scRNAseq (Fig. 2A), expressed as a proportion of all quality-controlled events in Fig. 1C.

F. Gating strategy for identifying $T_{RH}$ cells and $i.v.^-$ Treg among $CD4^+$ T cells by spectral flow cytometry.

G. Bar graph showing normalized enrichment score of selected GSEA pathways generated from ranked differential expressed genes comparing TRH populations between young and aged hosts at 30 d.p.i.

Data in (D) and (G) were analyzed by GSEA, with significance indicated as *, p < 0.05; **, p < 0.01; ***, p < 0.001; ****, p < 0.0001.

**Supplementary Fig. 3 Characterization of B cell clusters in young and aged hosts.**

A. Dot plot illustrating the defining features of each B cell cluster.

B. Kinetics of B cells quantified from the scRNAseq data presented in Fig. 3A, expressed as a proportion of all events passing quality control in Fig. 1C.

In (B), each dot's size represents the percentage of events in each sample expressing the indicated gene, and the color intensity reflects the average expression level.

**Supplementary Fig. 4 Characterization of mononuclear phagocytes in young and aged hosts.**

(A-B). UMAP of Mononuclear phagocyte (MNP) identified in Fig. 1C. Cells were colored subsequent re-clustering (A), and cell cycle stage (B).

C. Dot plot showing selected features used to define dendritic cell (DC) clusters. Dot size and color intensity represents the percentage of cells and average expression level, respectively, in given genes (rows) and cluster (columns).

D. Kinetics of DCs quantified from the scRNAseq data presented in (A), expressed as a proportion of the total quality-controlled events in Fig. 1C.

E. Dot plot illustrating selected features used to characterize MNPs by flow cytometry. Dot size and color intensity represents the percentage of cells and average expression level, respectively, in given cell types (rows) and genes (columns).

F. Kinetics of DCs (gating strategy shown in Fig. S1D) in the lung, quantified by flow cytometry.

G. Gating strategy for DC populations.

bioRxiv preprint doi: https://doi.org/10.64898/2025.12.15.694321; this version posted December 17, 2025. The copyright holder for this preprint (which was not certified by peer review) is the author/funder, who has granted bioRxiv a license to display the preprint in perpetuity. It is made available under aCC-BY 4.0 International license.

(H-J). Bar graphs displaying the quantification of DC subsets at ~60 d.p.i., including cDC1 (H), $CD11b^+PD\text{-}L1^-$ DCs (I), and $CD11b^+PD\text{-}L1^+$ DCs (J).

Data were pooled from at least three animals per time point (F-J). In (F), each dot represents the mean value for a sample, with color-filled error bars. Statistical analysis was performed using two-way ANOVA. In (H-J), results were pooled from two experiments; each dot represents one animal, and statistical analysis was performed using an unpaired Student's t test with Welch's correction. Significance levels regarding the impact of age as a source of variation are shown as: ns, $p \geq 0.05$; *, $p < 0.05$; **, $p < 0.01$; ***, $p < 0.001$; ****, $p < 0.0001$.

**Supplementary Fig. 5 Characterization of monocytes and macrophages in young and aged hosts.**

A. Dot plot displaying selected features used to define subsets of monocytes and macrophages. Dot size and color intensity represents the percentage of cells and average expression level, respectively, in given clusters (rows) and genes (columns).

B. UMAP plots showing the distribution of monocyte/macrophage clusters across with respect to individual samples.

C. Gating strategy for identifying macrophage populations by flow cytometry.

(D-E). Kinetics of alveolar macrophages (AMs) in the lung, quantified by CYTEK. AM subsets include $CD11b^+$ AMs (D, left) and $CD11b^-$ AMs (D, right). Within the $CD11b^-$ AM population, Siglec-$F^{hi}$ (as shown in Fig. 4E) and Siglec-$F^{lo}$ AMs were quantified (E).

(F-G). Bar graphs showing normalized enrichment score of selected GSEA pathways generated from ranked differential expressed genes comparing comparing IM1 (F) or IM2 (G) between young and aged hosts.

Data were pooled from at least three animals per data point (D-E). Each dot represents the mean value per sample, with error bars filled in color. Statistical analysis was performed using two-way ANOVA. Data in (F-G) were analyzed by GSEA. Significance levels are indicated as: ns, $p \geq 0.05$; *, $p < 0.05$; **, $p < 0.01$; ***, $p < 0.001$; ****, $p < 0.0001$.

**Supplementary Fig. 6 Exuberant type I and type II interferon signaling synergistically drives chronic sequelae in aged lungs.**

A. Dot plot displaying *Ifng* expression by defined cell types (Fig. 1C). Dot size and color represents the percentage of cells and average expression level, respectively, in given cell

bioRxiv preprint doi: https://doi.org/10.64898/2025.12.15.694321; this version posted December 17, 2025. The copyright holder for this preprint (which was not certified by peer review) is the author/funder, who has granted bioRxiv a license to display the preprint in perpetuity. It is made available under aCC-BY 4.0 International license.

types (rows) and genes (columns).

B. Dot plot illustrating expression of type I IFN genes in defined cell types (Fig. 1C). Dot size and color represents the percentage of cells and average expression level, respectively, in given cell types (rows) and genes (columns).

C. Dot plot showing mRNA levels of type I and type II IFN receptors across defined cell types (Fig. 1C). Dot size and color represents the percentage of cells and average expression level, respectively, in given cell types (rows) and genes (columns).

D. Gating strategy for Tregs and $T_{RH}/T_{FH}$ cells.

E. Representative plots of Tregs in the lung and mLN for each experimental group.

F. Bar graph quantifying Tregs in the lung.

G. Bar graph quantifying Tregs in the mLN.

In (F, G), each dot represents one animal. Statistical analysis was performed using repeated measures (RM) one-way ANOVA with Geisser-Greenhouse correction and multiple comparisons. Significance is indicated as *, $p < 0.05$; **, $p < 0.01$; ***, $p < 0.001$; ****, $p < 0.0001$.

**Supplementary Fig. 7 Cell-cell interaction analyses reveal unique pathways in young and aged hosts, and potential interactions between IMs and CD4$^+$ T cells.**

A. Unique interactions identified in young or aged hosts at 0, 2, and 9 d.p.i.

B. Kinetics of *Rorc* (RORγt)$^+$ cells quantified from scRNAseq data (Fig. 2A), expressed as a proportion of all events passing quality control in Fig. 1C.

C. Dot plot showing selected features used to define subsets of *Rorc* (RORγt)$^+$ cells. Dot size and color represents the percentage of cells and average expression level, respectively, in given clusters (rows) and genes (columns).

D. Chord plot illustrating CCL signaling in young and aged hosts during the memory phase. Each arrow indicates an inferred ligand-receptor interaction. Each arrow originates from the signal-sending cell type and points toward the signal-receiving cell type. Arrow color represents the identity of the signal-sending cell, while arrow thickness reflects the inferred interaction strength.

E. Dot plot displaying ligands involved in CCL signaling within MNPs (Fig. S4D). Dot size and color represents the percentage of cells and average expression level, respectively, in given genes (rows) and cell types (columns).

(which was not certified by peer review) is the author/funder, who has granted bioRxiv a license to display the preprint in perpetuity. It is m
available under aCC-BY 4.0 International license.

**Supplementary Figure 1, Related to Figure 1**

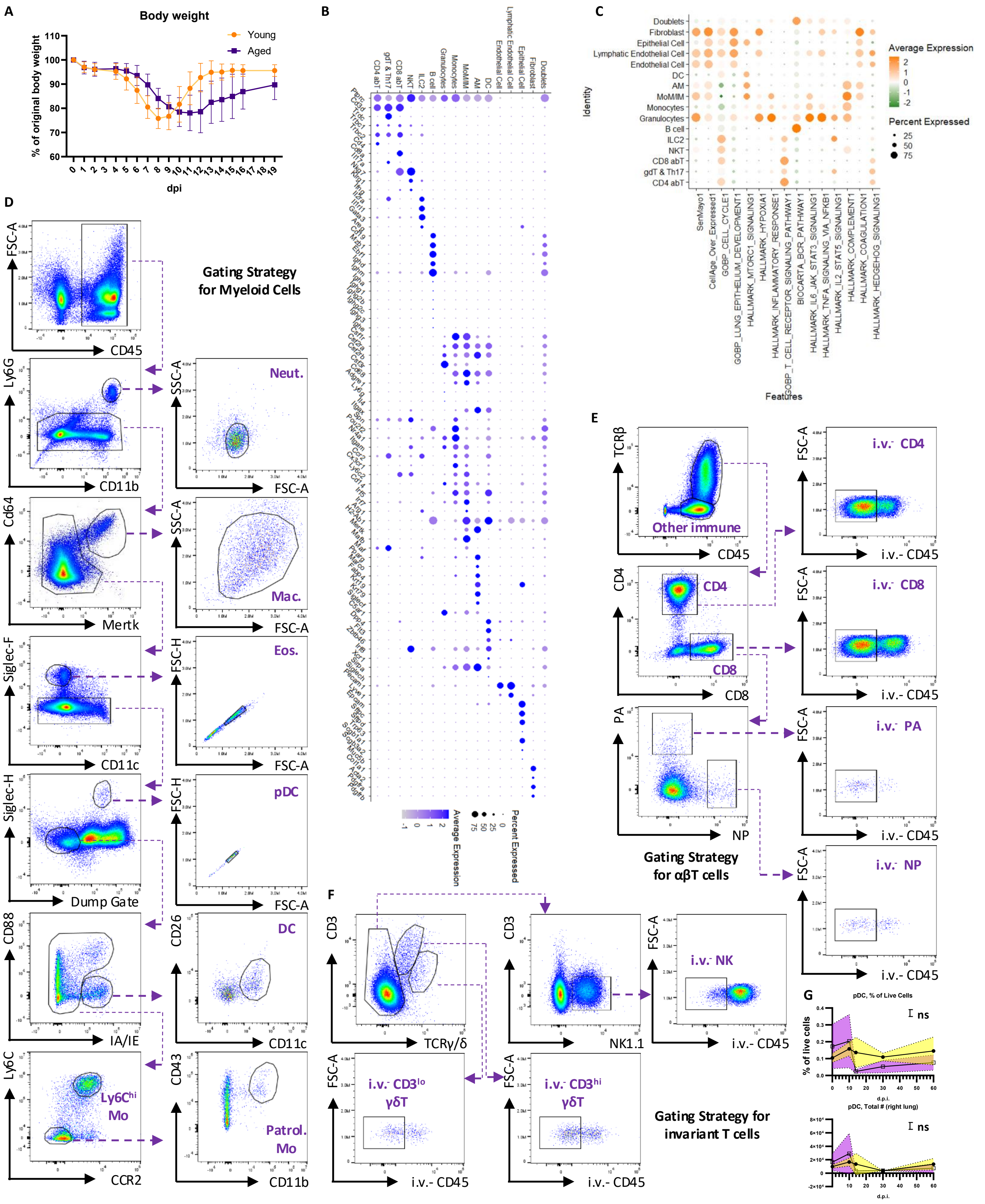

(which was not certified by peer review) is the author/funder, who has granted bioRxiv a license to display the preprint in perpetuity. It is m
available under aCC-BY 4.0 International license.

**Supplementary Figure 2, Related to Figure 2**

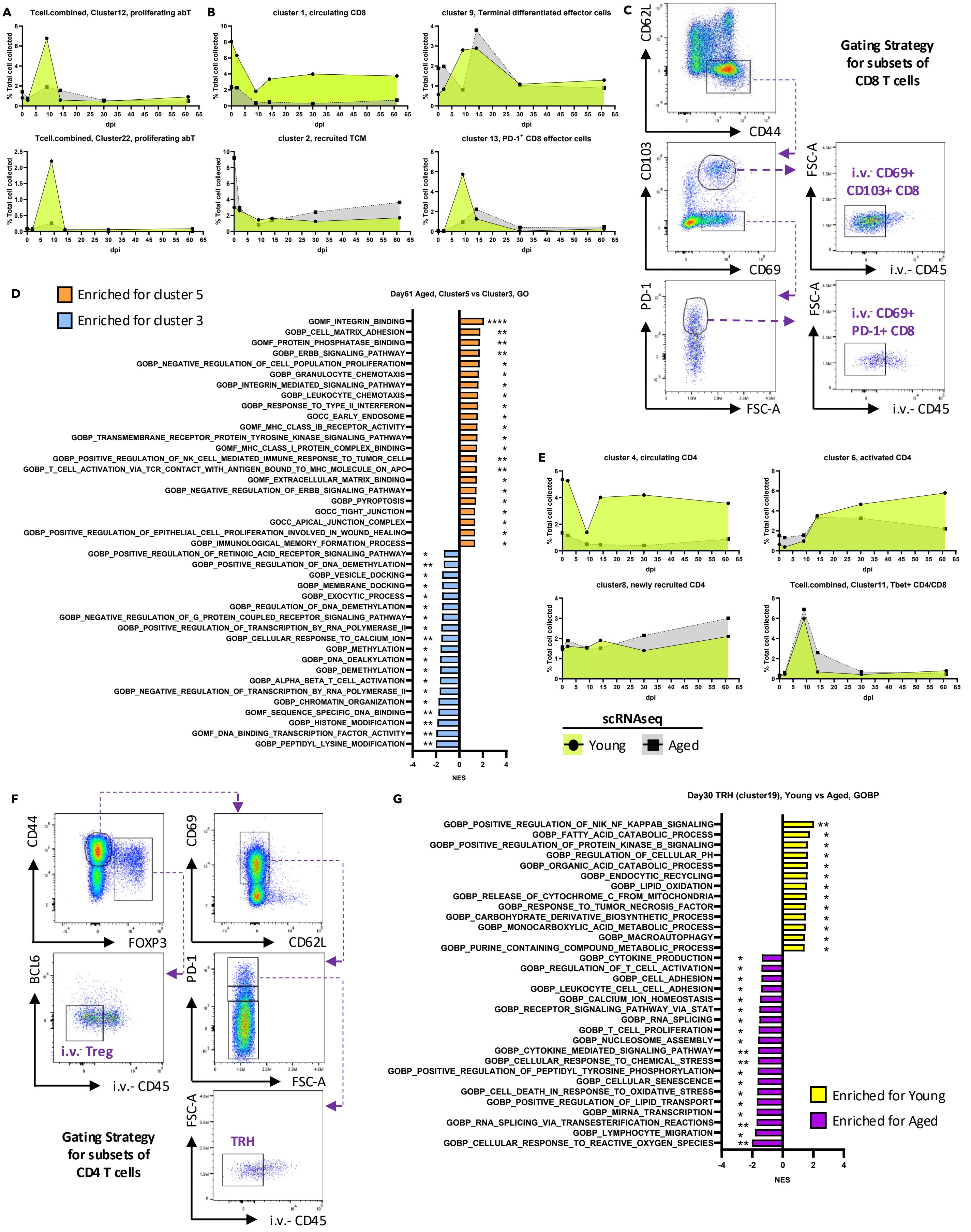

(which was not certified by peer review) is the author/funder, who has granted bioRxiv a license to display the preprint in perpetuity. It is m
available under aCC-BY 4.0 International license.

**Supplementary Figure 3, Related to Figure 2**

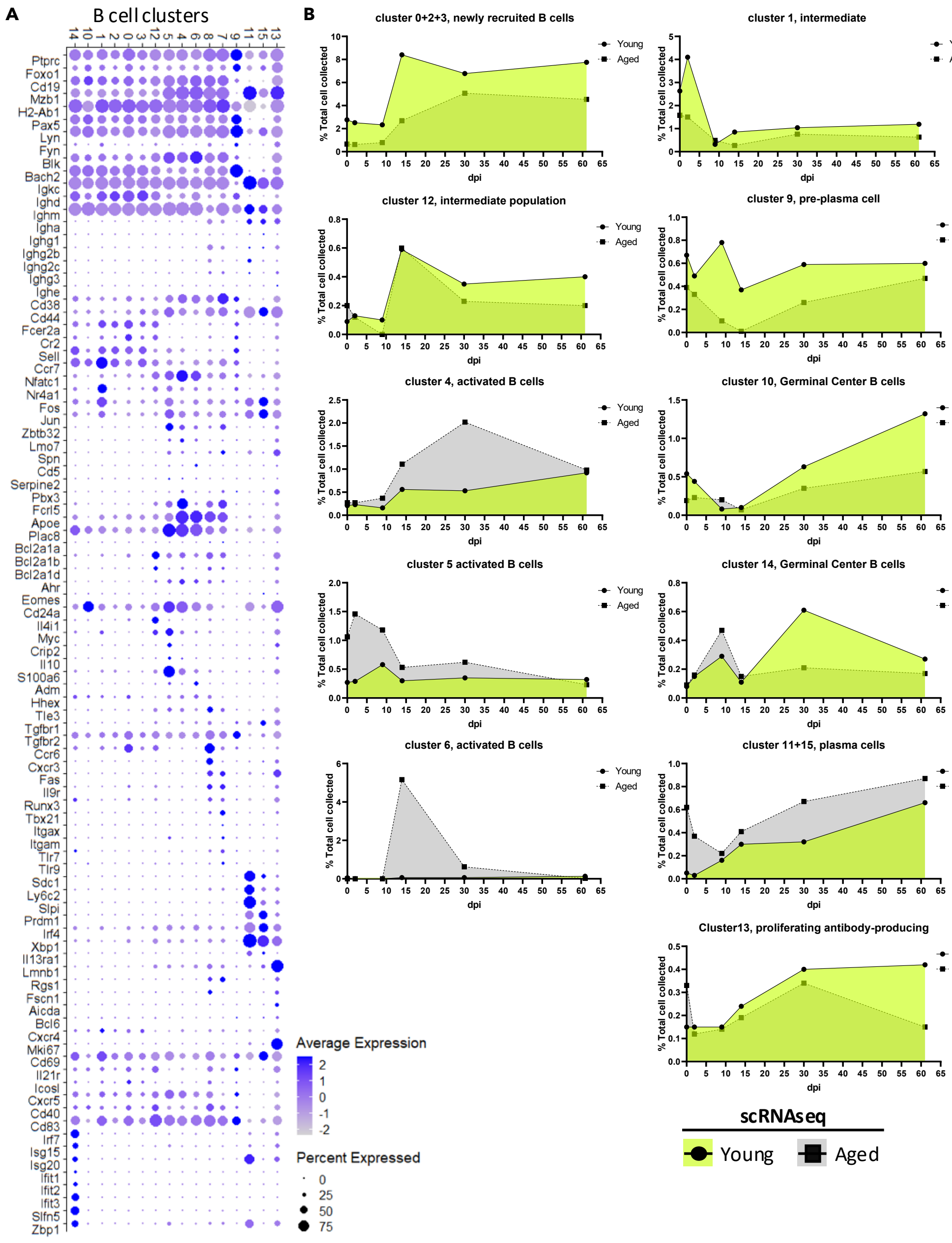

(which was not certified by peer review) is the author/funder, who has granted bioRxiv a license to display the preprint in perpetuity. It is m
available under aCC-BY 4.0 International license.

**Supplementary Figure 4, Related to Figure 4**

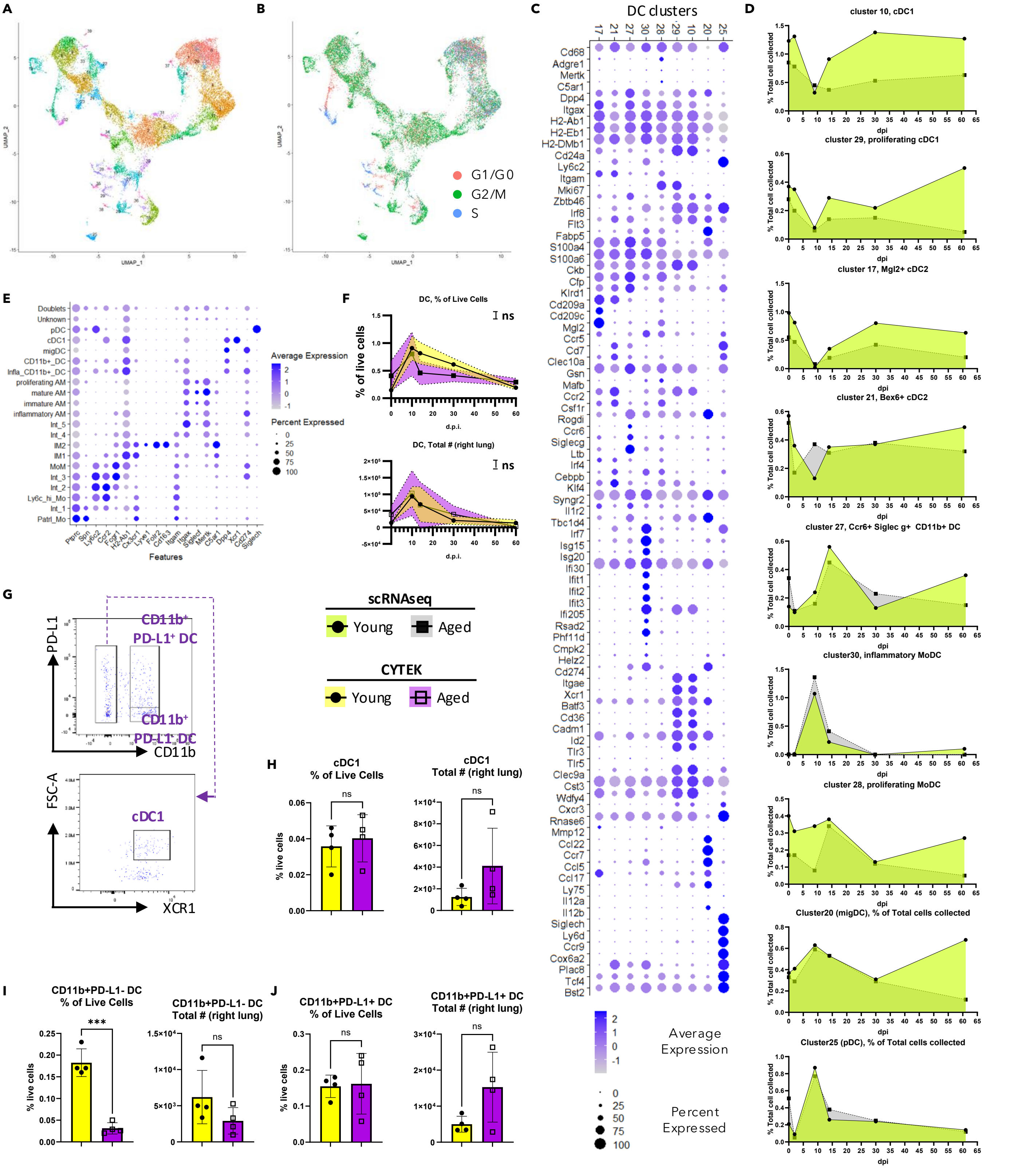

(which was not certified by peer review) is the author/funder, who has granted bioRxiv a license to display the preprint in perpetuity. It is m
available under aCC-BY 4.0 International license.

**Supplementary Figure 5, Related to Figure 4**

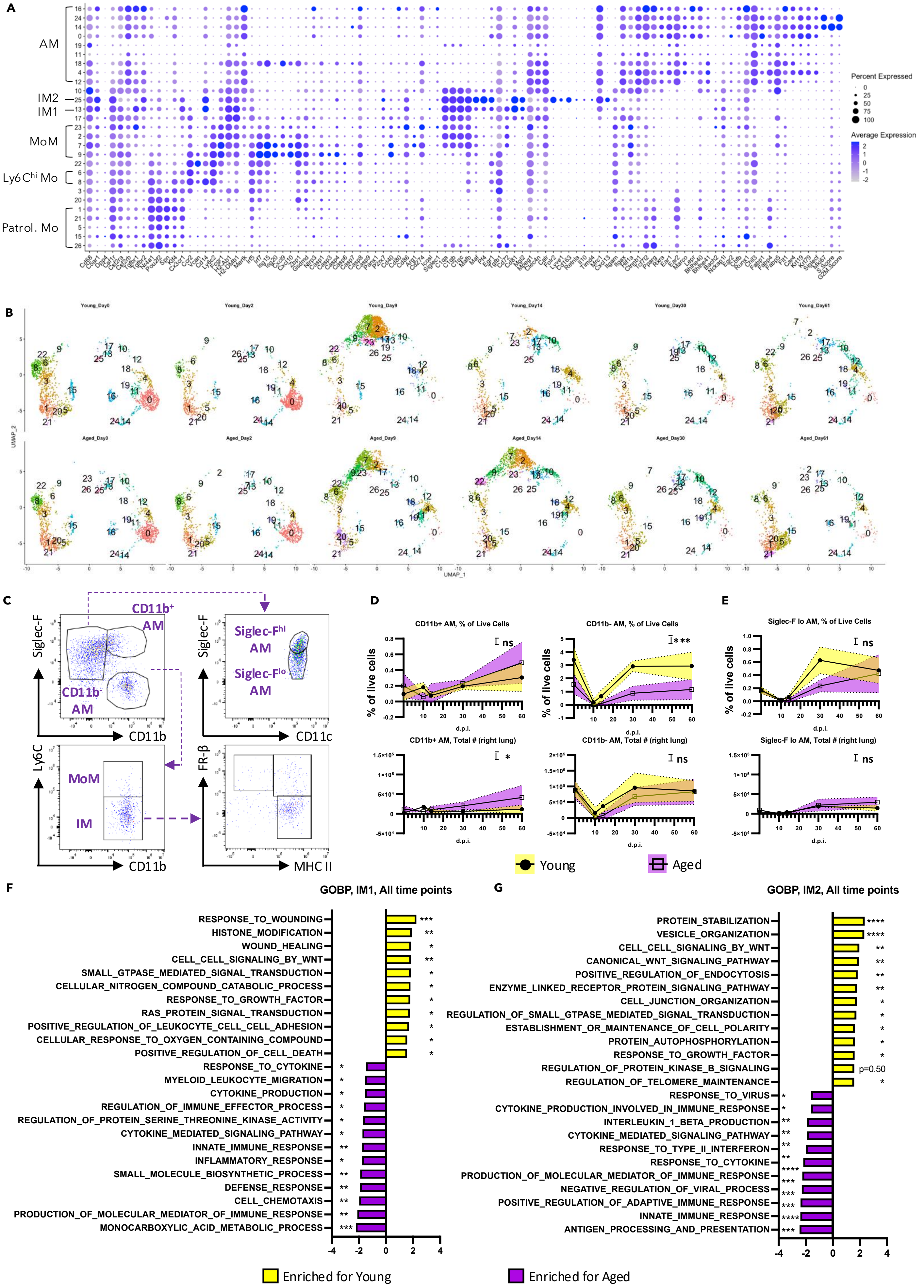

(which was not certified by peer review) is the author/funder, who has granted bioRxiv a license to display the preprint in perpetuity. It is m
available under aCC-BY 4.0 International license.

**Supplementary Figure 6, Related to Figure 5**

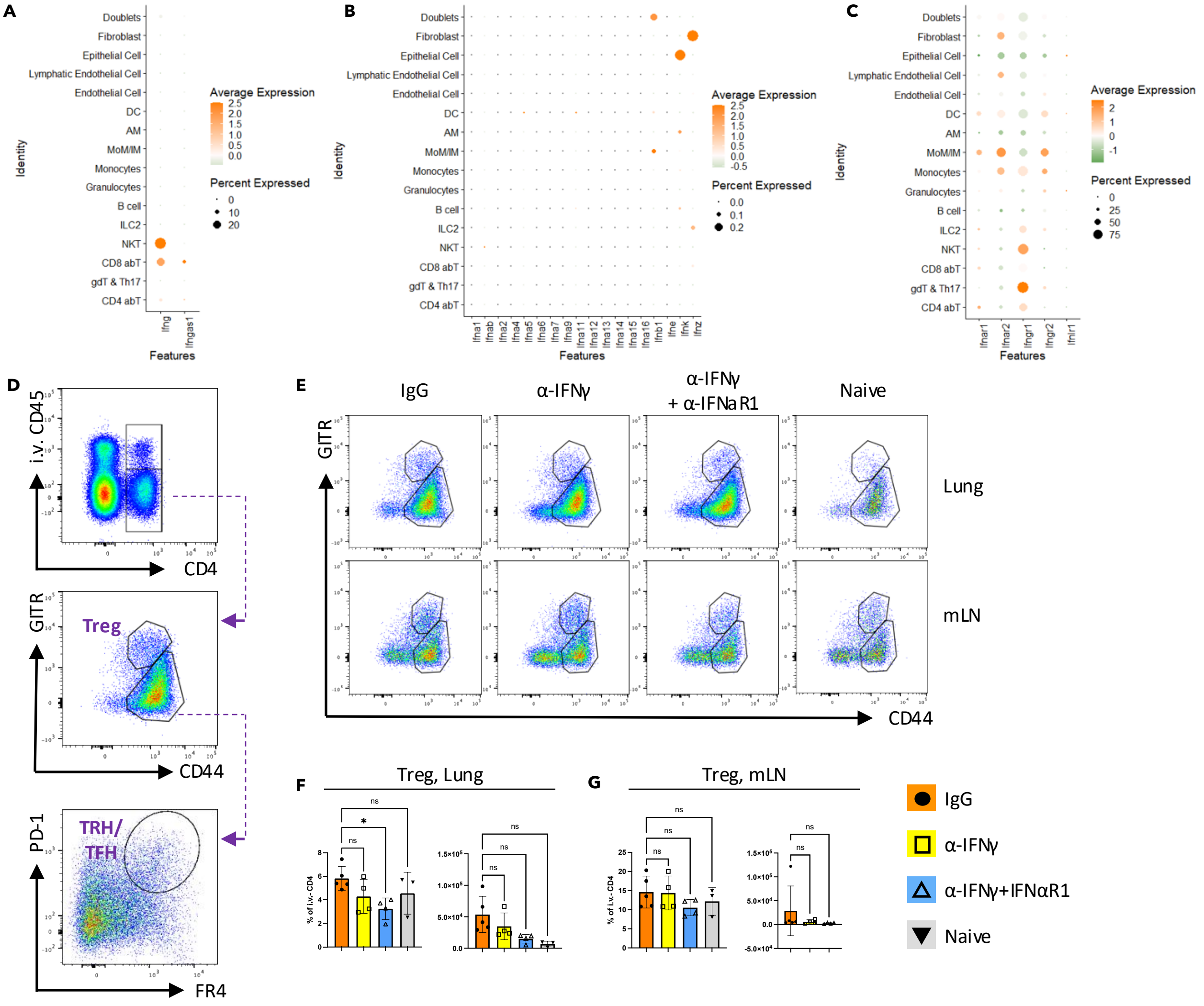

(which was not certified by peer review) is the author/funder, who has granted bioRxiv a license to display the preprint in perpetuity. It is m
available under aCC-BY 4.0 International license.

**Supplementary Figure 7, Related to Figure 6**

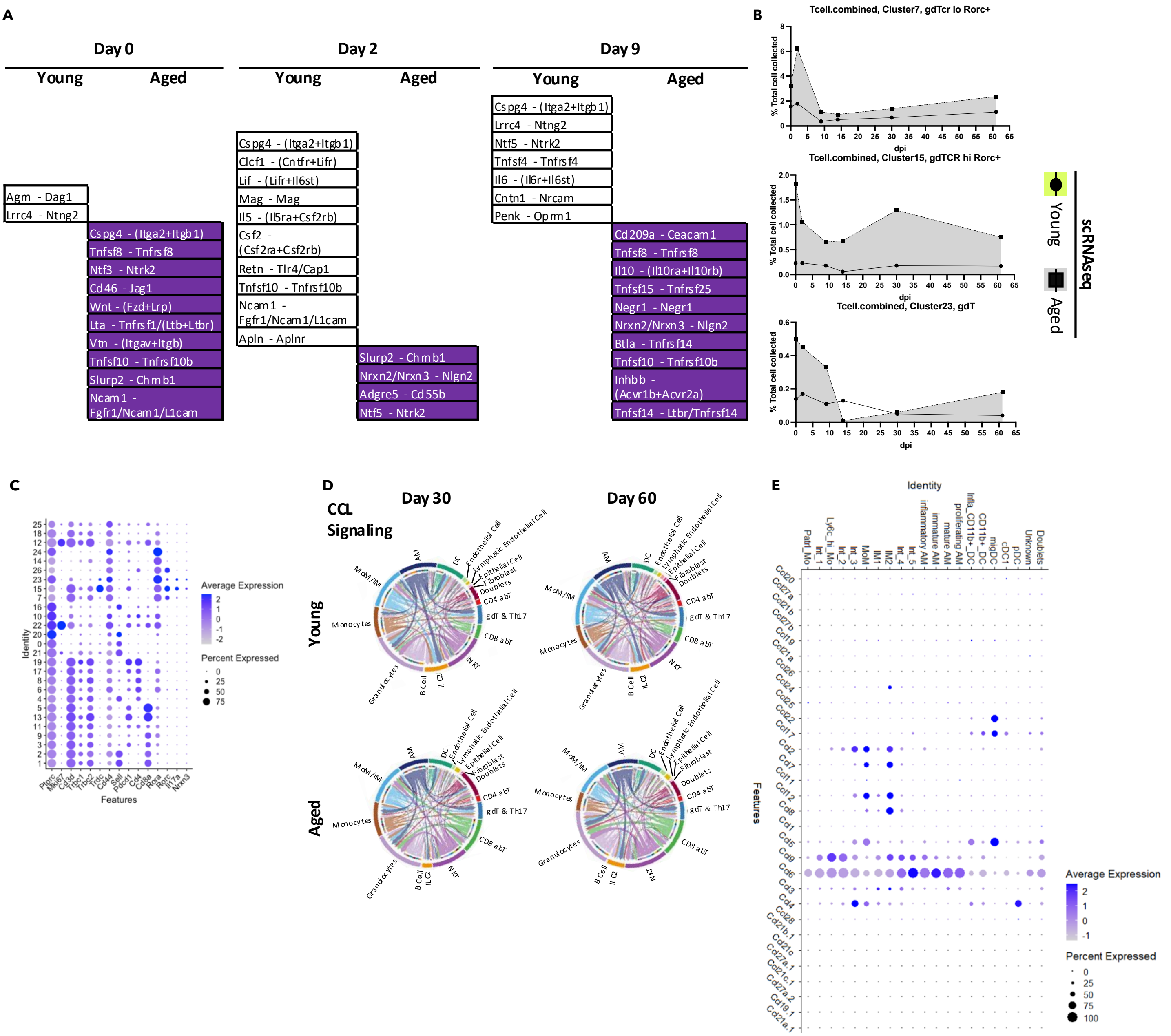

Supplement: Supplement 1 [file NIHPP2025.12.15.694321v1-supplement-1.pdf]
